# Extremophilic Exopolysaccharides: Biotechnologies and Wastewater Remediation

**DOI:** 10.3389/fmicb.2021.721365

**Published:** 2021-08-19

**Authors:** Aparna Banerjee, Shrabana Sarkar, Tanvi Govil, Patricio González-Faune, Gustavo Cabrera-Barjas, Rajib Bandopadhyay, David R. Salem, Rajesh K. Sani

**Affiliations:** ^1^Centro de investigación en Estudios Avanzados del Maule (CIEAM), Vicerrectoría de Investigación Y Posgrado, Universidad Católica del Maule, Talca, Chile; ^2^Centro de Biotecnología de los Recursos Naturales (CENBio), Facultad de Ciencias Agrarias Y Forestales, Universidad Católica del Maule, Talca, Chile; ^3^Department of Botany, UGC-Center of Advanced Study, The University of Burdwan, Golapbag, Burdwan, India; ^4^Department of Chemical and Biological Engineering, South Dakota Mines, Rapid City, SD, United States; ^5^Composite and Nanocomposite Advanced Manufacturing – Biomaterials Center, Rapid City, SD, United States; ^6^Escuela Ingeniería en Biotecnología, Facultad de Ciencias Agrarias Y Forestales, Universidad Católica del Maule, Talca, Chile; ^7^Unidad de Desarrollo Tecnológico (UDT), Universidad de Concepción, Coronel, Chile; ^8^Department of Materials and Metallurgical Engineering, South Dakota Mines, Rapid City, SD, United States; ^9^BuGReMeDEE Consortium, South Dakota School of Mines and Technology, Rapid City, SD, United States

**Keywords:** bioremediation, commercialization, environment, exopolysaccharide, extremophile

## Abstract

Various microorganisms thrive under extreme environments, like hot springs, hydrothermal vents, deep marine ecosystems, hyperacid lakes, acid mine drainage, high UV exposure, and more. To survive against the deleterious effect of these extreme circumstances, they form a network of biofilm where exopolysaccharides (EPSs) comprise a substantial part. The EPSs are often polyanionic due to different functional groups in their structural backbone, including uronic acids, sulfated units, and phosphate groups. Altogether, these chemical groups provide EPSs with a negative charge allowing them to (a) act as ligands toward dissolved cations as well as trace, and toxic metals; (b) be tolerant to the presence of salts, surfactants, and alpha-hydroxyl acids; and (c) interface the solubilization of hydrocarbons. Owing to their unique structural and functional characteristics, EPSs are anticipated to be utilized industrially to remediation of metals, crude oil, and hydrocarbons from contaminated wastewaters, mines, and oil spills. The biotechnological advantages of extremophilic EPSs are more diverse than traditional biopolymers. The present review aims at discussing the mechanisms and strategies for using EPSs from extremophiles in industries and environment bioremediation. Additionally, the potential of EPSs as fascinating biomaterials to mediate biogenic nanoparticles synthesis and treat multicomponent water contaminants is discussed.

## Introduction

Polysaccharides are biopolymers composed of monomeric sugars linked *via* glycosidic bonds and are the most abundant macromolecules on our earth. They are an omnipresent part of the living world, be it plants, animals, algae, fungi, bacteria, or archaea, and are known to play an essential role in maintaining their structural integrity and functionality (Casillo et al., 2018). Extracellular polysaccharides or exopolysaccharides (EPSs) are high molecular weight complex biopolymers that are majorly composed of sugars and include other non-carbohydrate organic substances, like proteins, lipids, humic substances, or extracellular DNA as their constituents (Rana and Upadhyay, 2020).

Naturally, EPS synthesized by microbes have a unique advantage over other polysaccharides isolated from plants (cellulose, mannan, starch, and pectin), animals (glycogen), and algae (agar, carrageenans, alginic acids, and alginates). The microbially derived EPS are produced and secreted by the hosts outside the cells, making EPS harvesting from the cell-free supernatant easy and cost-effective while avoiding the use of environmentally damaging cell lysing chemicals (Schmid et al., 2015). EPS synthesized by bacteria further offer shorter production times and ease of growth (Pereira et al., 2019). Since the chemical composition of the EPS determines its functional applications (Freitas et al., 2011), bacteria as the production hosts under controlled batch or continuous fermentation modes allow extraction of EPS with consistent, specific functional, and tailored features. By comparison, the plant and algal polysaccharide compositions more or less fluctuate and are readily influenced by climatic factors (Pereira et al., 2019).

Extremophilic microbes surviving under hostile environments purposely produce and surround themselves with a layer of EPS as a survival strategy (Merino et al., 2019; López-Ortega et al., 2021). EPS affords protection to the bacteria against the extreme conditions of temperature (Casillo et al., 2018), salinity (Isfahani et al., 2018), aridity, and desiccation (Knowles and Castenholz, 2008). The EPS matrix also protects the host bacterium against antimicrobial antibiotics and drugs (Merino et al., 2019; Wang et al., 2019). Above all, EPS is an inherent component of biofilms (Rossi and De Philippis, 2015) and helps the bacterium in surface attachment, colonization (Janczarek et al., 2015), and nutrient uptake (Kalpana et al., 2018). Until now, reports on a vast number of bacterial EPSs are available in the literature, and the aspects related to their biosynthesis, structure, and functions during conditions of stress have been analyzed in depth. More recently, the application of EPS in high-value market niches, such as food, biomedical, and cosmeceuticals (Banerjee and Bandopadhyay, 2016; Halder et al., 2017; Laubach et al., 2020), where other polysaccharides fall short of compliance with the necessary degree of purity and biocompatibility, are gaining ground. Indeed, the biodegradability, biocompatibility, non-toxicity, and chemical functionality of the EPS are the essential factors behind their possible commercial applications (Wang et al., 2019).

Microbially produced EPSs are non-toxic, biocompatible, and biodegradable polymers that have found outstanding applications in different industries (Siddharth et al., 2021). In biomedical industries, their utility as conjugates for drug and vaccine-controlled delivery is well-recognized (Moscovici, 2015). Some EPSs, such as sphingan, gellan gum, welan, rhamsan, and diutan from marine isolates, are known for their tremendous sliminess or high viscosity, making them useful as a transparent, thickening agent, stabilizer, and binder, particularly in the food and cosmetics industries (Fialho et al., 2008; ANS et al., 2018). Other potential EPS applications, especially from halophiles, are related to their usage as emulsifiers and surfactants (Chikkanna et al., 2018). These applications include bioremediating oil-contaminated fields and *in-situ* degradation of long-chain *n*-alkanes and petroleum hydrocarbons (Poli et al., 2010). The EPS that some extremophiles produce is rich in charged anionic ligands, such as uronic acid residues (glucuronic acid and galacturonic acid) and sulfates. Together, both these groups present EPS with a negative charge that poses them as ligands for scavenging toxic and heavy metals from wastes (Giudice et al., 2020). Further, EPSs produced by the extremophiles offer great potential in wastewater treatment, intending to solve present-day water scarcity. Recognizing EPS outstanding ecological and commercial value, extremophilic bacterial EPSs have been projected to be a cost-effective, sustainable, and straightforward alternative to economical bioremediation of the environment. In the present review, the biotechnological advantages of extremophilic EPSs, their functional mechanisms, and strategies for their utilization in wastewater bioremediation are discussed, along with their role in some recent nano-biotechnological advances.

## Biotechnological Advantages of Extremophilic Eps Over Traditional Biopolymers

The primary biotechnological advantages of EPS arise from the ability of these complex polysaccharides to stabilize the host against a harsh external environment while maintaining their structural integrity. More recently, the aspects of commercial applications of extremophilic EPS in biotechnology have started to earn attention (Parwani et al., 2021). In the psychrophilic strains, EPS generates a thin sheet of water to thwart extracellular ice from destroying the cell surface and has been shown to act as a cryoprotectant against freezing (Krembs et al., 2002; Poli et al., 2010; Rice et al., 2015; López-Ortega et al., 2021). There is evidence that EPS in at least one of these strains can form a pseudohelicoidal structure that may prevent the formation of the local tetrahedral order of the water molecules in the first hydration shell and thwart extracellular ice from destroying the cell surface (Casillo et al., 2018). Nichols et al. (2005) characterized cold-stimulated EPS produced by deep-sea microbial isolates belonging to the genus *Pseudoalteromonas*, *Shewanella*, *Polaribacter*, and *Flavobacterium*. The isolated EPSs contained neutral sugars (e.g., glucose, fructose, mannose, and galactose) as the main components. Distinctively, the psychrophilic EPSs were rich in uronic acid residues and contained some sulfated units. Other research groups have reported similar findings for the EPS composition in psychrophilic microbes (Raguénès et al., 1997, 2003), where they have reported the presence of phosphate groups. Altogether, these chemical groups provide EPS with a negative charge allowing it to act as ligands toward dissolved cations and trace metals, such as iron (Fe), zinc (Zn), copper (Cu), cadmium (Cd), and cobalt (Co; Zhang et al., 2017). The affinity of EPS toward iron (Fe^+3^) is of critical importance in the marine environment where 99% of iron is ligated to organic compounds and is generally unavailable to primary producers such as phytoplankton (Scharek et al., 1997; Wu et al., 2001) and diatoms (Krembs et al., 2002). The power of EPS from Antarctica isolates to allow their growth under high concentrations of cadmium and mercury (up to 7,500ppm) has been reported by Caruso et al. (2018). Thus, psychrophilic EPS in the marine ecology is a boon that can enhance primary production, besides playing a cryoprotective function in the cold ecosystem. Such EPSs, rich in anionic groups, are likewise of biotechnology interest in bioremediation and wastewater treatment plants for bio-adsorption of toxic metals from the wastes (Giudice et al., 2020).

In yet another application, an α-mannan rich EPS from a new Arctic permafrost isolate, *Sphingobacterium* sp. IITKGP-BTPF3 has demonstrated antioxidant activities *via* scavenging of superoxide anions and reducing nitric oxide production (Chatterjee et al., 2018). The activity of EPS to activate macrophages (Zhang et al., 2018) and improve cell viabilities (Yu et al., 2016) supports the role EPS’s can play in biomedical fields. Indeed, the role of EPSs from Antarctica isolates on the biogeochemical cycling of calcium and iron in the marine environment, aiding the production and sequestration of dissolved and particulate organic material, aggregation of dust particulates on the surface of the glaciers, and as an essential source of carbon for microorganisms in the food chain have also been recently documented (Bhaskar and Bhosle, 2005; Nagar et al., 2021).

In other studies, sphingan EPSs, such as gellan gum, welan, rhamsan, and diutan, have been found in marine isolates belonging to the genus *Sphingomonas*, which lack uronic acids in them (Denner et al., 2001; Huang et al., 2009; Tala et al., 2013). Of these, gellan gum produced by *Sphingomonas elodea* ATCC 31461 has been exploited commercially (West, 2021). It has been known to produce a clear, viscous gel/solution that is insensitive to heat and acids, and hence gellan gum can be used as a fluid for oil well drillings (Fialho et al., 2008). Gellan gum has been considered a suitable concentrating agent for jams and confectionery products (Widyaningrum and Meindrawan, 2020). Also, the commercially available form of gellan gum, “Gelrite,” is a good substitute for agar in the culturing of microbes at high temperatures (thermophiles) and also in plant tissue and animal cultures (Shungu et al., 1983). Gelrite also has potential applications for encapsulation experiments in the laboratory (Li et al., 2016). Lately, Sphingan’s utility in the medical field is increasingly recognized for applications as complete as tissue regeneration, bone repair, dental fillings, and allergy relief (Petre, 2019). Gellan based beads and films have also being investigated for drug delivery (Fialho et al., 2008). One of the other sphingan EPSs, rhamsan, has been used in agriculture for suspending pesticides and fertilizers since they are stable in salt solutions (Li et al., 2016). Likewise, the xanthan-like EPS produced by bacterium groups, such as *Xanthomonas campestris* (Barua et al., 2016), *Alteromonas infernus* (Akoumany et al., 2019), and *Paenibacillus tarimensis* (Boukhelata et al., 2019) could find application as a food-stabilizer in sauces (Widyaningrum and Meindrawan, 2020). Dextran’s application as a viscosifier, stabilizer, conditioner, and emulsifier of dairy products, including its use in bread doughs to increase airiness and softness, has been well-recognized (Bohn, 1958). The recent review articles by Casillo et al. (2018), Widyaningrum and Meindrawan (2020), list and summarize the main application of EPSs derived from marine sources in the food industry.

As an extremophilic group, Halophiles are found in hypersaline environments, such as saline lakes, salt pans, salt marshes, or saline soils, where the salt concentration typically ranges between 0.2 and 5.1M (1–30%; Amoozegar et al., 2019). The EPSs isolated from halophiles have common usage as emulsifiers (Gan et al., 2020). Mauran, an EPS produced by hypersaline microbes from the genus *Alteromonas* (Mata et al., 2008) and *Halomonas* (Chikkanna et al., 2018), has been reported more efficient than commercial surfactants. The EPS they produce is rich in anionic ligands (especially sulfate; Ruiz-Ruiz et al., 2011) and has a high pseudoplastic flow with the capability to form a barrier between two immiscible liquids. This positions EPS as an effective emulsifier, and it allows the macromolecule to interface the solubilization of hydrocarbons to enhance the biodegradation of oil-spill zones (Poli et al., 2010; Llamas et al., 2012; de Jesús Paniagua-Michel et al., 2014). Several studies now demonstrate that halophilic EPSs are competitive candidates for *in situ* degradation of long-chain *n*-alkanes and petroleum hydrocarbons (Cappello et al., 2012, 2014; Gutierrez et al., 2013; Fan et al., 2020).

In recent years, increased demand for thermophilic EPSs has been observed in pharmaceutical, food, and other industries, largely dependent on their slow thermal degradation (González-Faune et al., 2021). Antiviral and immune-modulating activities have been demonstrated for EPSs extracted from thermophilic *Bacillus licheniformis* (Arena et al., 2006) and *Geobacillus thermodenitrificans* (Arena et al., 2009). Results indicate that immunological disorders can be treated with thermophilic EPSs. As for their application in biotechnology, EPS from thermophiles provides fermentative advantages, such as reduced growth time, improved nutrients, higher oxygen mass transfer rates, and resistance to toxic inhibitors released during the depolymerization of lignocellulosic materials (Nicolaus et al., 2010; López-Ortega et al., 2021). EPS from thermophiles, such as *Streptococcus thermophilus*, have also been shown to improve product viscosity and texture (Xiong et al., 2021). Recent evidence accumulates to demonstrate that even thermophilic EPS are decorated with sulfated anionic groups with high arabinose and xylose content. Such thermostable EPS’s can further expand the potential range of activities and potency of EPS- derived health-promoting agents (Sardari et al., 2017). A sulfate rich EPS purified from a thermophilic bacterium *Anoxybacillus pushchinoensis* G11 with antibiofilm, and antitumor (lung and colon) activities are an example to support their application (Genc et al., 2021).

The application of DeinoPol, an EPS from an extreme radiation-resistant *Deinococcus radiodurans*, as a stress protectant against UV radiation and reactive oxygen species has also been demonstrated by Lin et al. (2020). The results indicated that DeinoPol significantly delayed the mutational and death rate in *D. radiodurans* exposed to a high concentration of hydrogen peroxidase and ϒ-radiation. Hence, DeinoPol has remarkable potential in skincare and pharmaceutical industry applications as a reliable and attractive oxidant scavenger. Not explored yet, but DeinoPol may prove effective for remediating radionuclides, such as uranium, plutonium, thorium, radium, etc., from the contaminated sites (Lin et al., 2020). Supplementary Figure 1 summarizes some potential roles that EPS can play in biotechnology and related areas.

## Application of Extremophilic Eps For Environment Bioremediation

**Figure 1:**
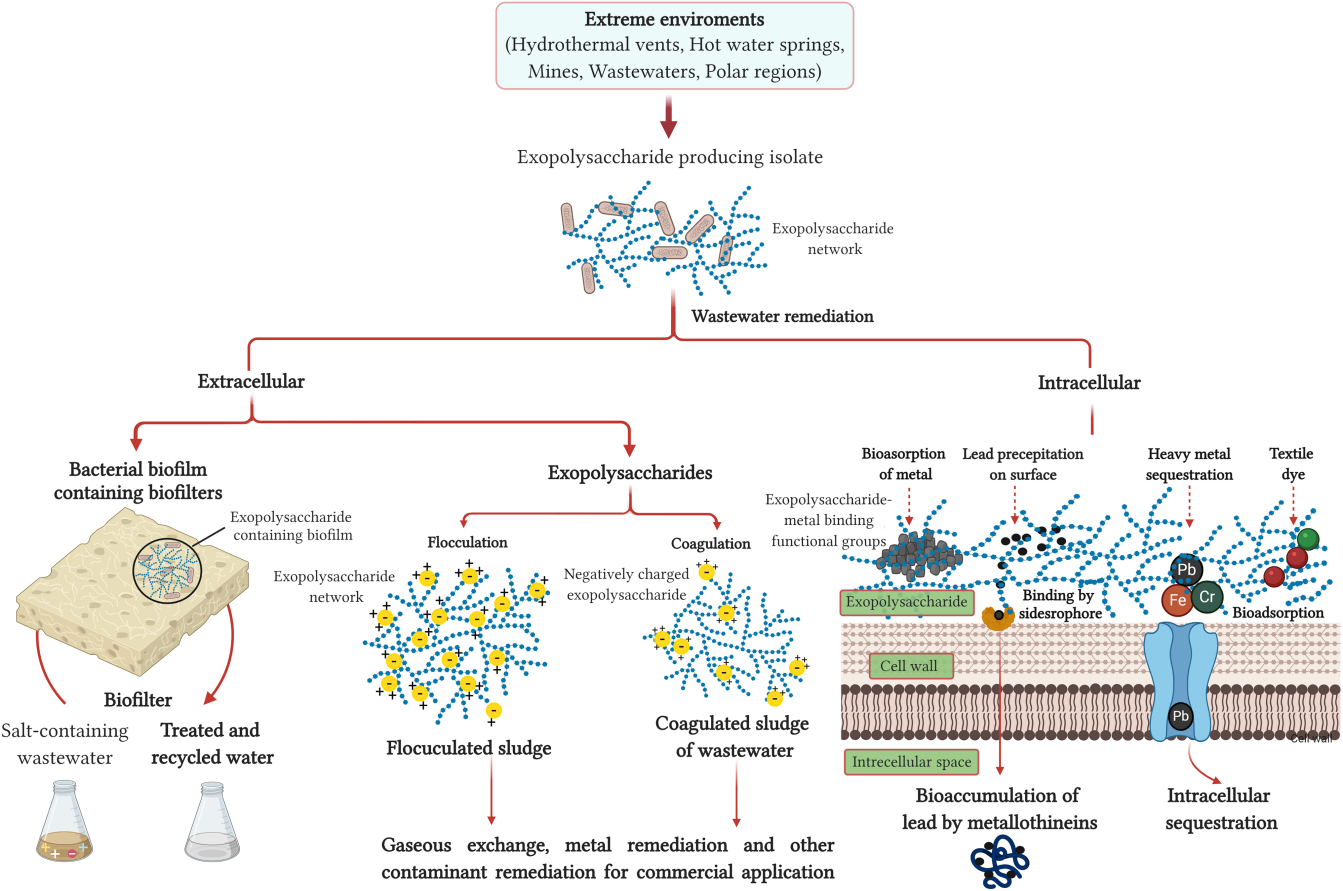
The generalized concept of exopolysaccharide (EPS) produced by extremophilic bacteria and its different wastewater treatment strategies.

Fast industrialization has remarkably accelerated the discharge of toxic wastewater and solid waste materials, which are the prime sources of environmental contamination, with prolonged effects on our planet. Conventional wastewater treatment strategies, like mixing, sedimentation, filtration, or chlorine disinfection, are primarily expensive and produce secondary pollutants (Sarkar et al., 2017; Crini and Lichtfouse, 2019; Syafiuddin and Fulazzaky, 2021). By contrast, biological treatments are generally reported to employ anaerobic sludge bed or expanded granular sludge bed reactors and aerobic thermophilic bioreactors (Lettinga et al., 1993; LaPara and Alleman, 1999). Both these treatment strategies are particularly well-suited to eliminating recalcitrant and toxic components, but the biological treatment produces no secondary pollutants. Activated sludge flocs have been intensively studied and applied for the past 100years in contrast to anaerobic granules that have been investigated since the 1980s and aerobic granules that have only received attention since the late 1990s (Ding et al., 2015). However, the drawbacks of the biological anaerobic and aerobic treatments are excess sludge production and less aggregate generation, respectively (Lettinga et al., 1993; LaPara and Alleman, 1999). To fight against these inadequacies, one of the emerging biological waste treatment processes comprises polysaccharide biopolymers/EPSs that are efficient, environment friendly, and economical to be produced by bacteria (Zhou et al., 2010). Some reports earlier have detailed the applicability of bacterial EPS biopolymer in wastewater treatment, many of which are produced by extremophilic bacteria (Freitas et al., 2011). The generalized concept of extremophilic bacterial EPS and its utility in the treatment of different waste components like salts, heavy metals, textile dye, and ionic wastes are demonstrated in Figure 1. The application of EPS in bioremediation arises because most of the EPS (especially from extremophiles) are polyanionic from the presence of uronic acids or inorganic phosphate or sulfate residues (Poli et al., 2010). Principal strategies for using extremophilic bacterial EPSs in environment bioremediation include the usage of bacterial EPS-containing biofilms as (a) biofilters, (b) flocculation, coagulation, and emulsification of sludge, oil, or dye components, and (c) biosorption and bioaccumulation of heavy metals.

### EPS as Supporting Materials for Biofilters

Effective removal of nutrients in the wastewater treatment plants *via* biological means requires microbial strains with efficient carbon or nutrients (mainly nitrogen and phosphorus) removal properties to be immobilized in agar beads or as biofilms (Guarin and Pagilla, 2021). To date, several biofilm-based reactor configurations (for example, trickle bed, packed bed, fluidized bed, airlift suspensions, upflow anaerobic sludge blanket, rotating biofilm, etc.) have been developed and tested (Mendez and Lema, 1992; Nicolella et al., 2000; Qureshi et al., 2005), where the filter comprises EPS material surrounding a colony of indigenous microbial communities that perform at least one of the essential functions of the filtration process (Hammes et al., 2011). This immobilization process essentially enables the cells to be retained, propagate, and maintain their activity. Studies suggest that the dynamics and properties of the EPS produced within the system are critical for biofilm growth and have direct implications on the efficiency of the biofiltration process (Andersson, 2009). Microbes retained and covered within the EPS layer establish themselves as biofilms, biochemically oxidize the biodegradable organic matter, and make *in situ* filtering of the wastewater a possibility. Other specific advantages of EPS enclosed biofilm life are the metabolic cooperation for substrate exchange, the presence of microniches with a gradient of oxygen and nutrient concentrations, and enhanced gene transfer rates. These facilities within the biofilm create a favorable environment for a good variety of complex differentiated microbial populations to attain the desired metabolic or biochemical reaction rates for the enhanced degradation of the recalcitrant or biodegradable organic and inorganic compounds in the wastewater (Andersson, 2009; Guarin and Pagilla, 2021).

Andersson (2009) tested the ability of two denitrifying organisms, *Comamonas denitrificans* and *Brachymonas denitrificans*, as biofilters immobilized on 20 different low-cost carriers, and underlined the importance of EPS and its composition of detectably developed biofilms with the denitrification activity. The author reported similar findings for the phosphorus removal activity of *Acinetobacter calcoaceticus*, *Acinetobacter iwoffi*, and *Aeromonas hydrophila*, where the EPS deficient strains failed to form proper three-dimensionally structured biofilm and had a low overall phosphorus removal activity (Andersson, 2009). In another study, the algal biofilm reactors (with EPS producing *Chlorella vulgaris* as the biofilter) integrated with wastewater treatment have been shown to remove more than 90% of nitrogen content and 80% of phosphorus content in the effluent from the primary treatment of municipal wastewater (Hoh et al., 2016). In a recent study by Gallardo-Rodríguez et al. (2019), biofilters with natural fibers and living microbial cells were evaluated for continuous inflow of Pb^2+^ (325mg/day). Interestingly, the biofilm accelerated the adsorption of Pb^2+^ at 72h, and the maximum adsorption capacity was observed to be 48.75mg/g at neutral pH (Gallardo-Rodríguez et al., 2019). These studies and parallel observations published in the literature claim that EPSs support the entrapping of organic materials and microorganisms, hence obliquely boosting the straining properties of the filter (Hammes et al., 2011). Besides, EPS can decrease the harmful impacts of the toxic substances on the microbial bio-filters and can also be utilized as the carbon source or energy source, for the microbes in the absence of nutrients (Maurya and Raj, 2020). Indeed, in the microbes isolated from the wastewater treatment plants, genes encoding EPS production have already been identified (Kim et al., 2019). Gelation and multivalent cation binding property of EPS extracted from activated sludge at different stages or operational steps of the wastewater treatment plants has been already discussed in review papers. Ding et al., 2015). Based on these promising observations, the emerging scope and role of EPS as an efficient, low-cost, low- maintenance supporting material for wastewater treatment in biofilm-mediated remediation technology deserve the pursuit of more in-depth studies and a greater understanding of their function and properties.

### Flocculation, Coagulation, and Emulsification of Sludge, Oil, or Dye Components

The second majorly studied approach for the EPS-mediated wastewater treatment process is flocculation, which generally targets the textile, paper, and tannery industry’s wastewater components. These industries are responsible for more than 20% of global water pollution alone (Hasanbeigi and Price, 2015). They have recently been found to release large amounts of dye-containing alkaline waste effluents (Samsami et al., 2020). There are several reports on the use of bacterial EPS for this kind of wastewater remediation. One of the first studies related to this area was psychrotolerant bacterial EPS from an Antarctic sea-ice bacterium *Pseudoalteromonas* sp. Bsi20310. It has shown enhanced coagulation capacity against a synthetic dye, reactive brilliant red X-3B, in the presence of ferric chloride; 150mg/L of the bacterial EPS with 55mg/L ferric chlorides enhanced the decolorization process up to 90% (Zhou et al., 2010). This EPS was of high molecular weight with carbon backbones having different added functional groups like carboxyl and hydroxyl that probably interact with the ferric ions, favoring floc formation in the coagulation process (Zhou et al., 2010). Treatment obstacles with wastewater containing azo textile dye are the water’s high salinity and the presence of different heavy metals. In this context, EPS produced by salt-tolerant *Halomonas* sp. AAD6 was found effective to treat brackish industrial wastewater by flocculation in lower concentrations (100mg/L doses of EPS) and lower residual turbidity (Sam et al., 2011). Two principal characteristics of bacterial EPSs are the bridging and the electrostatic effects, which help coagulation-mediated wastewater treatment (Bratby, 2016). The charge density of EPS helps to configure the treatment recipe of the solution. An increase in molecular weight, net charge density, and electrostatic repulsion between two charged units increases the viscosity of the polymeric solution in the treatment (Bratby, 2016). In this context, another example is the marine bacterium *Aliiglaciecola lipolytica*, which is reported to degrade nearly 45% of azo dye waste primarily by adsorbing it onto EPSs. Notably, protein-like humic-acid containing tightly bound EPS (TB-EPS) produced by *A. lipolytica*, can decrease the electrostatic force that increases the cell surface’s hydrophobicity, leading to improved adsorption (Wang et al., 2020). It had been already explained earlier that increased protein content of EPS leads to greater hydrophobicity, which causes superior flocculation activity (Shi et al., 2017). In connection to it, EPS produced by *Bacillus* sp. YP03 (140μg/ml, pH 7.5) in the presence of trivalent aluminum ion demonstrated coagulation followed by flocculation. It showed 47 and 89% reductions of chemical oxygen demand and total suspended solids, respectively in local municipal wastewater treatment (Kanmani and Yuvapriya, 2018). This mechanism also contributed to dye adsorption and cell self-flocculation that may be considered a promising technique for wastewater treatment (Wang et al., 2020). According to Sam et al. (2011), the bacterial EPSs, being a naturally available, biodegradable, biocompatible, and non-toxic flocculant, can be ideally used to treat this kind of textile, paper, and tannery wastewater.

From an earlier report, alkaliphilic bacterium *Cronobacter sakazakii* isolated from oil-contaminated wastewater produced biosurfactant compounds, decorated with reducing sugars, protein, uronic acid residues, and sulfate groups. These compounds showed emulsifying activity (emulsification index ranges from 60 to 100% for 1mg/ml biosurfactant concentration) against aromatic and aliphatic hydrocarbons (Jain et al., 2012). The biosurfactant EPS also demonstrated pseudoplastic rheology. Interestingly, the presence of protein and uronic acid makes this biosurfactant a good emulsifier for toxic heavy metals, oils, and hydrocarbons, thus improving the bioremediation of contaminated wastewater (Jain et al., 2012). Allen et al. (2004) and Fida et al. (2017) reported similar biosurfactant properties, where EPS produced by *Thauera* sp. demonstrated valuable multifunctionality; oil emulsification, bio-plugging, and biodegradation of acetone and isopropyl alcohol in wastewater. To add, EPS produced from a marine bacterium *Pseudomonas furukawaii* PPS-19 (isolated from the oil-polluted site in India) was found to emulsify and degrade 89.52% degradation of crude oil within 5days. These properties demonstrated the potential of EPS-forming microbes as bio-emulsifier for their application in the bioremediation of petroleum-polluted sites (Vandana and Das, 2021). Overall, it may be assumed that extremophilic bacterial EPSs can be potentially used to treat a wide range of components present in wastewater due to their unique structural configurations (Table 1). To get a better overview of the industrialization of extremophilic bacterial EPS-mediated wastewater treatment, an online patent analysis tool, Relecura, was used.^1^ It may of interest to note that while there have been momentous studies in this area for the last decade, only seven patents have been filed for this technique, out of which a total of six were from China and only one, the first patent, was reported by the European Patent Office (EPO) in the year of 1992. Flocculation and discoloration of the wastewater were found to be the basis of all the patents.

**Table 1:** Main sources and characteristics of EPS produced by extremophilic bacteria in wastewater treatment-related processes in last 5years.

Biosorbent	Biosorbent properties	Type of effluent/isolation source	Operating conditions	Target pollutant	Other components	Maximum sorption capacity (q_e_, mg/g), Removal efficiency (Re, %), and others	References
*Geobacillus stearothermophilus* DG1	Hetero-EPS Glucose/Galactose/Fucose/Mannose/Glucuroic acid	High-temperature wastewater from an oil refinery in Dagang, China	Batch, 0.5% molasses, 0.3% (NH_4_)_2_HPO_3,_ and 0.2% NaNO_3_	*n*-alkanes, *n*-alkyl cycloalkanes, and heteroatom compounds	NA	NA	Li et al., 2021
*Bacillus cereus* KMS3–1	–	Metal amended conditions	Batch mode, 100mg/L for each metal	Cd^2+^ Cu^2+^ Pb^2+^	NA	q_e_ 54.05 Cd(II) q_e_ 71.42 Cu(II) q_e_ 78.74 Pb(II)	Mathivanan et al., 2021
*Parapedobacter* sp. ISTM3	α-D-glucose, mannofuranoseanhydro-fructose	Mixed solution of heavy metals	Batch, 20mg/L each heavy metal	Cr^6+^	Zn^2+^, Cu^2+^, Pb^2+^, Cr^6+^, Fe^2+^, and Cd^2+^	q_e_ 33.783 Cr^6+^ Re 95.1% Cr^6+^ 79.5 Pb^2+^ 76.1 Cd^2+^	Tyagi et al., 2020
*Bacillus licheniformis* DM-1	Proteoglycan type EPS	Mature reservoir in Dagang oil field, China	30g/300ml crude oil	*n*-alkanes (C12–C36^+^)	NA	Re 70% C16–C22 Re 81.33% C18	Fan et al., 2020
*Halomonas elongata* S6	Highly anionic EPS	Sehline Sebkha Salt Lake, Tunisia	0.01% flocculating agent	Ca^2+^	Kaolin solution	NA	Joulak et al., 2020a
*Halomonas* *Smyrnensis* K2	Hetero-EPS K2 mannose (66.69%), glucose (19.54%), galactose (13.77%)	Soil samples from solar saltern of Kerkennah, Tunisia	0.1ml of 2mM FeCl_2_·4H_2_O and 0.2ml of 5mM ferrozine	Fe^2+^	NA	q_e_ 31.1 Fe^2+^	Joulak et al., 2020b
*Bacillus cereus* KMS3-1	Heteropolymeric mannose (73.51%), glucose (17.87%), predominant monosaccharide xylose (2.18%), rhamnose (6.49%)	Multi metal-containing surface sediment from Southeast coast of India	Flocculation on 40mg/L of EPS, pH 7.0 Metal tolerance in 5mg/L multi-metal (Cd, Cu, Pb, and Zn) concentration	Cd^2+^ Cu^2+^ Pb^2+^ Zn^2+^	NA	88.35% flocculation 100mg/L Cd^2+^ 225mg/L Cu^2+^ 1,750mg/L Pb^2+^ 125mg/L Zn^2+^	Krishnamurthy et al., 2020
*Klebsiella oxytoca DSM 29614*	EPS-Fe^0^NPs complex	Mimicked groundwaters	Batch metal solution 5mg/L	As^5+^	As^3+^	Re 87–95% As^5+^ 45–61% As^3+^	Casentini et al., 2019
*Streptomyces* sp. CuOff24	EPS, arabinose 42.6%, galactose 28.6%, glucose 22.4%, mannose 12.4%	Radionuclide Solution	Batch100mg/L SrCl_2_+100mg/L CaCl_2_	Sr^2+^	CaCl_2_ 100mg/L	q_e_ 46.3Sr^2+^ Re 93.3%Sr^2+^	Kamala et al., 2020
*Exiguobacterium profundum* PT2	EPS biofilm Uronic acids and fatty acids	Artificial waste-water effluent	Batch10m MNa_2_HAsO_4_. 7H_2_O	As^5+^	NA	q_e_ 29.4As^5+^	Saba et al., 2018
*Sphingomonas sp. MKIV*	Glucose galacturonic acid	Ionic liquids (municipal wastewater treatment) plant (Sewerage Board of Limassol – Amathus, Moni, Cyprus)	100mg/L of [BMIM][MeSO_4_] and [*n*-Bu4N][I]	Imidazoliu, pyridinium-, and ammonium based ionic liquids [Py][CF3SO3] [1-4PPy][Cl] [BMIM][Br] [BMIM][MeSO_4_] [*n*-Bu4N][I] [*n*-Bu4N][PF6]	NA	Re 91% [BMIM][MeSO_4_] Re 87% [*n*-Bu4N][I]	Koutinas et al., 2019
*Bacillus cereus* VK1	l-Arabinose, d-Glucose, Lactose, Xylose, d-Galactose	Metal solution	Batch 1.5mMHgCl_2_	Hg^2+^	NA	q_e_ 295.5Hg^2+^ Re 100% Hg^2+^	Kalpana et al., 2018
*Halomonas nitroreducens* WB1	Anionic EPS glucose mannose galactose	Agnikundahot spring of Bakreshwar, India	200ml 0.1mM solution of the cations (Cd, Co, Cu, Pb, Ni, and Zn)	Pb>Zn>Cd>Cu>Co>Ni	NA	Partial affinity for Pb was 182mg/g, In uni-metal solution affinity 263mg/g for Pb	Chikkanna et al., 2018
*Acidithiobacillus thiooxidans*	Pel Exopolysaccharide	Acidic environment (ATCC 19377)	NA	Cu^2+^	NA	NA	Díaz et al., 2018
*Lactobacillus plantarum*-605	Mannose, Glucose and Galactose (molar ratio 28:36:36), –Acetyl, –SO_3_ and –PO_4_ groups	Mixed solution of heavy metals	Batch 1g/L each heavy metal NO_3_^−^ salt Dye solution	Pb^2+^ Cd^2+^ Cu^2+^ Methylene blue	NA	q_e_ 2,327 Pb^2+^ 548 Cd^2+^ 935 Cu^2+^ 2,800 Methylene blue Re 80% Pb^2+^, 59% Cd^2+^, 57% Cu^2+^, 75% Methylene blue	Li et al., 2017
*Deinococcus radiodurans* R1	NA	Mixed solution of heavy metals	Batch metal solution NO_3_^−^ salt 1mg/L	Co^2+^ Ni^2+^	25mM Ca^2+^	Re 35% Co^2+^ 25% Ni^2+^	Shukla and Rao, 2017
*Thauera* sp. TK001	NA	Shallow, low temperature American Petroleum field [Medicine Hat Glauconitic C (MHGC) and American Petroleum Institute (API)]	25mM acetone or 22.2mM IPA	Isopropyl alcohol (IPA) and acetone	NA	NA	Fida et al., 2017
*Pseudomonas* sp. W6	Metallophilic EPS (Amino, cyanide, hydroxyl, carbonyl and carboxyl ligands containing)	Hot water spring from North–East India	1mM/L PbNO_3_	Pb^2+^	NA	Re 65% synthetic effluent	Kalita and Joshi, 2017

*NA, not available*.

During the last decade, plenty of studies were devoted to the isolation and characterization of extremophile bacterial strains and their EPSs, or analyzing their biosorption properties and their bioremediation potential (Krishnamurthy et al., 2020; Joulak et al., 2020a). However, most of the studies have in common that they were characterized for bioremediation at a lab scale. There is only scarce information about the scaling-up potential of bacterial EPS to promote their industrial utilization as biosorbents. In this sense, further studies devoted to large-scale EPS application on wastewater remediation are needed to establish the process operation conditions like pH, temperature, ionic strength, bio-sorbent doses, agitation speed, batch, or continuous operation (Park et al., 2010; Kim et al., 2020). Conversely, studies testing industrial effluent samples are needed to identify the right strategy for bio-sorbent application at a large scale, e.g., using the encapsulated bacterial strain, using the biomass of dead microorganisms, or using bacterial EPS alone (Xie et al., 2020). Moreover, it is necessary to have a clear pollutant recovery strategy throughout the flocculation and separation processes, column elution, metal-biomass burning, or a disposal/reuse strategy (e.g., life-cycle assessment studies and catalyst use of metal-EPS/biomass complex; Alipanah et al., 2020). Finally, a cost assessment of the microbial biomass/EPS production (Nwosu et al., 2019) and the whole biosorption process would be required. This is because various highly available, low-cost bio-sorbents from nature (e.g., macro/microalgae, mosses, plants, and animals), and others obtained as industrial by-products, can be used for the same purposes but may have the drawback of seasonal scarcity, target pollutant non-selectivity, and structure variability between batches that would limit their application. Based on the above discussion, an excellent future for EPS application as a bio-sorbent is foreseeable. However, gaps need to be filled between lab-scale and industrial applications to bring more of these products into the market.

### Bio-Sorption and Bioaccumulation of Heavy Metals

Mining industries are primarily responsible for massive metal waste generation (Gupta and Diwan, 2017; Gayathri et al., 2021). For metal biosorption, bacterial EPSs have already demonstrated significant capability to salvage the environment from hazardous heavy metal-containing wastewater, the basis of which is polyanionic bacterial EPSs interacting with positively charged metal ions or ionic exchange mechanism (Joulak et al., 2020b). Size, ionic nature, and charge activity of single or multiple metal ion solutions regulate its interaction with anionic bacterial EPSs (Gupta and Diwan, 2017; Zhao et al., 2020).

Indeed, in the case of large-scale water purification experiments, columns packed with highly anionic EPS-yielding halophilic bacterial strains, either immobilized or attached with suitable carriers, are utilized as an adsorbent filler material (Gupta and Diwan, 2017). For better results, pH, temperature, inorganic-organic ligands, ionic strength, and other physio-biochemical parameters need to be strictly controlled. Biosorption by EPS also helps bacteria to use inorganic ions as their metabolic element (Gupta and Diwan, 2017). An earlier study by Llamas et al. (2010) on *Salipiger mucosus* showed bio removal activity of toxic metals (15.7mg Cu, 43.5mg Pb, and 8.7mg Co chelation by 1g of EPS) from wastewater and polluted environments. The possible mechanism was attributed to the acetyl groups present in the EPS conveying more electron-donating groups into the vicinity of the binding site, which allows the larger metal ions to bind more firmly (Llamas et al., 2010). Anionic EPSs are reported to prefer binding with metal cations containing a sizeable ionic radius. For its excellent metal ion chelating activity, this halophilic biopolymer produced by *S. mucosus* A3^T^ was further efficiently used to treat industrial effluents containing heavy metals (Llamas et al., 2012). In this type of application, anionic EPS produced by halophilic bacteria *Halomonas almeriensis* manifested the adsorption capacity of different heavy metals like Pb (24.5mg sorption/1g EPS), Cu (19.2mg sorption/1g EPS), and Co (10mg sorption/1g EPS; Llamas et al., 2012). Generally, hypersalinity pressure is deemed a limiting factor that affects the survivability and efficiency of the microorganisms in the wastewater treatment plant. However, for the treatment of wastewaters with high salt concentrations, EPS is a crucial compound that some hosts use to scavenge Na^+^ permitting their endurance in high NaCl stress (Zeng et al., 2016). EPS’s protective response to Na^+^ in biofilm reactors has been demonstrated in the literature (Zhang et al., 2011; Zheng et al., 2011; Nunkaew et al., 2015). The ability of EPS to mitigate stress on microbes from heavy metals and osmotic salts such as Na^+^ has prospects to even alleviate the stress of salt on plants growing in saline and/or toxic environments (Nunkaew et al., 2015). Thus, the addition of purified EPS or the microbes producing EPS can help improve the quality of biofertilizers and improve the tolerance of plants to salinity/toxicity.

Since thermophilic EPSs are rich in neutral carbohydrates, especially rhamnose, and may contain uronic acids, as in EPS from psychrophiles, they have potential in industrial applications such as bioleaching (Zhang et al., 2019), immobilization of metals, such as cadmium (Arena et al., 2006) and chromium, and bio emulsification (Zheng et al., 2011). For instance, a Chromium (VI)-reducing *Bacillus* sp. isolated from tannery activated sludge was proved to be able to entirely reduce 50mg/L of Cr (VI) within 24h under aerobic conditions (Zhu et al., 2019). In another example, EPS-producing thermophilic *Pseudomonas* sp. W6 isolated from an Indian hot spring was shown to biosorb up to 1mM of lead (Pb), with an accompanying removal of approximately 65% of Pb from synthetic wastewater (Kalita and Joshi, 2017). In order to ameliorate the toxic effects of Pb, bacteria are already reported to produce EPS, which functions through biosorption, metal chelation using siderophores, and metallothionein production (Mitra et al., 2021). Therefore, bacterial EPS can be employed as an advantageous alternative to the conventional physical and chemical method of Pb remediation from industrial wastewater (Mitra et al., 2021). Further, a few other bacteria like *Lactobacillus plantarum*-605 and *Deinococcus radiodurans* R1 have even shown sorption efficiency for a wide number of heavy metals in NO^3−^ salt (Pb^2+^, Cd^2+^, Cu^2+^, Co^2+^, and Ni^2+^) as well as synthetic dyes (methylene blue) from a mixed solution of heavy metals (Li et al., 2017; Shukla and Rao, 2017). From another report, it has been found that functional groups (C=O, O–H, CH, C–O, and C–C=O) of EPS produced by *Bacillus cereus* KMS3-1 interact with metal ions to assist the detoxification process (Mathivanan et al., 2021). Here, the heavy metal (including Cadmium) removal efficiency (up to 48%) of EPS from psychrophilic microorganisms has been described, too (López-Ortega et al., 2021).

Another potential application for wastewater treatments mediated by bacterial EPS, which has recently been identified, is removing ionic liquids. These liquids are generally rich in organic/inorganic anions and nitrogen-containing cationic aromatic rings, causing acute toxicity to the environment. In one study, EPS produced by *Sphingomonas* sp. MKIV has been reported to remove nearly 90% of the noxious ionic liquids from the waste effluent (Koutinas et al., 2019). In another recent study, heteropolymeric EPS from *Streptomyces* sp. CuOff24 showed more than 93% removal efficiency of Sr^2+^ from radionuclide solution (Koutinas et al., 2019).

Additionally, metallic minerals (e.g., uranium and arsenate) and organic pollutants (e.g., dibenzothiophene) present in the wastewater were reported to be effectively treated with the help of electroactive bacteria like *Shewanella oneidensis* (sorption followed by reduction of U(VI)) and *Pseudomonas putida* (reduction of arsenate and biotransformation of dibenzothiophene). The redox property of heme-mediated protein-containing electrobacterial EPSs helps transporting electrons to generate electricity in waste materials (Li et al., 2016).

A further instance of EPS-mediated metal bioleaching has been reported by an EPS isolated from an acidophilic sulphur-oxidizing bacterium *Acidithiobacillus thiooxidans* (Díaz et al., 2018). The development of biofilm and the cell adherence to metallic ores promoted by the production of EPSs supported the bioleaching activity. Due to this attribute, the isolated acidophilic bacterium was reportedly applied in an industrial mining area for wastewater bioremediation (Díaz et al., 2018). Similar bioleaching activity for metal sulphides from natural mineral substrates such as pyrite has been reported for EPS extracted from thermoacidophilic archaeon *Acidianus* sp. DSM 29099 (Zhang et al., 2019), as well as from thermophilic *Acidithiobacillus caldus* (Huang et al., 2019). EPS produced by *Exiguobacterium profundum* PT2 has interestingly shown arsenic biosorption (~30mg/g arsenic sorption capacity) in the presence of 10mM Na_2_HAsO_4_.7H_2_O from artificial wastewater effluent (Saba et al., 2018). Previously, it was assumed that heavy metals remain in their toxic form within microbial systems. However, later it was detailed that heavy metals induced the microbial metabolic pathways in different ways. Active (bioleaching, bioaccumulation, and reduction of toxic metals) or passive (biosorption) uptake of these heavy metals assist in biological interactions to transform them into a less harmful or immobilized form and prevent intrusion into the bio-system (Gupta and Diwan, 2017; Haldar and Ghosh, 2020).

Hence, extremophilic EPSs may be projected as a cost-effective, sustainable, and simple alternative for the environment’s economical bioremediation, especially heavy metals. Their production is achievable on a commercial scale, and a projection suggests that just with the use of EPS, the concentration of heavy metals from the ecosystem can be diminished from parts per million (ppm) to parts per billion (ppb; Mohite et al., 2017). Moreover, Deschatre et al. (2015) has given a brief overview of EPS as an efficient bio sorbent of silver, a finding that can be explored further as a potential approach for mining some precious metals from their respective deposits.

A recent review by Kranthi et al. (2018), presents a comprehensive and elaborated assessment of the efficiency of EPS from different microorganisms to control heavy metal contamination and deal with environment maintenance and human health (Kranthi et al., 2018). Besides, literature is also abundant discussing the role that extremophiles (Supplementary Figure 2) can play in the environmental remediation of metal and organic pollutants (Peeples, 2014; Giovanella et al., 2020; Kaushik et al., 2021), xenobiotic compounds (Jeong and Choi, 2020; Shukla and Singh, 2020), radionucleotides (Marques, 2018), plastics and various agrochemicals (Kour et al., 2021), and synthetic pollutants (Bhatt et al., 2021).

## Recent Nanobiotechnology-Based Advances Involving Extremophilic Epss in Wastewater Bioremediation

Owing to its multidisciplinary nature, nano-biotechnology is increasingly impacting many areas of physics, chemistry, and biology. Apart from its application in drug delivery, biosensors, vaccine development, and genetic engineering, the potential role of nano-biotechnology in wastewater treatment is emerging. Biogenic nanoparticles appear to be promising candidates for wastewater bioremediation, among which biopolymeric metallic nanomaterials are of great scientific interest (Sarkar et al., 2020; Russo et al., 2021). The high surface area to volume ratio of nanoscale adsorbent materials bestows increased catalytic activity, due to which nano-adsorbents are presently capturing the spotlight in the field of wastewater treatment (Pereira et al., 2015). However, biogenic metal nanoparticles have certain negative aspects, such as extensive reduction time, complex downstream processing, and aggregate formation. To address these problems, EPS biopolymers can act as both reducing and capping agents in metal nanoparticle synthesis (Sathiyanarayanan et al., 2017). Having superior competence to synchronize with the metal ions, bacterial EPSs can be an effective alternative for treating wastewaters, which primarily includes environmental metal wastes, pharmaceutical wastes, and wastes from the textile industry and treatment of bactericidal agents.

In EPS biopolymers, reducing groups and the presence of charged functional groups, which help bind the polymer with other charged moieties such as metal ions and permit effective treatment of metal contaminants in wastewater (Durán et al., 2011). Therefore, bacterial EPS is often incorporated in the synthesis of nanoparticles to reduce the toxic heavy metals in the metal-contaminated environmental wastes. For example, natural weathering of selenium rich rock, mining, and irrigation release selenium, including its most toxic species, selenite (SeO_3_^2−^), into the environment. However, reducing groups in the bacterial EPSs results in the reduction and environmental detoxification of selenite in the form of elemental selenium nanoparticles (Zhang et al., 2020). There are other reports of bacterial EPS-mediated reduction and immobilization of metal ions to form nanoparticles. For instance, psychrotrophic EPS isolated from Arctic glacier bacterium *Pseudomonas* sp. PAMC 28620 exhibited superior metal removal activity against Fe^2+^ ions (more than 99%), making it an effective biosorbent for wastewater treatment (Sathiyanarayanan et al., 2016). In another report, EPS isolated from marine *Pseudomonas aeruginosa* JP-11 was used to synthesize spherical cadmium sulfide (CdS) nanoparticles within a size range of 20–40nm (Gahlawat and Choudhury, 2019). CdS NPs incorporated into xanthate-functionalized EPS from *P. aeruginosa* JP-11 could better adsorb the cadmium ions from metal-contaminated wastewater (88.66%) compared with the functionalized EPS alone (80.81%) and the pristine EPS (61.88%; Raj et al., 2016). Aspects of EPS-metal nano complex as a biosorbent for wastewater bioremediation have been well addressed by Sathiyanarayanan et al. (2016). Sulfated mauran (MR), one of the most studied extremophilic bacterial EPSs because of its fascinating rheological properties, attracts metal ions to form stable nanomaterials due to uronic acid (Sathiyanarayanan et al., 2017). MR isolated from a moderately halophilic bacterium *Halomonas maura* produced thin-uniform nanofibers (120nm) by electrospinning using a homogenous solution of poly-vinyl alcohol. These MR nanofibers were reported to treat metal-containing environmental wastewater (Raveendran et al., 2013). Also, novel magnetic nanocomposite was effectively blended *via* the co-precipitation of Fe_3_O_4_ nanoparticles [iron (III) chloride and iron (II) sulfate] with EPS derived from the microalga *C. vulgaris*. These magnetic EPS nanoparticles were effective in removing 91% of PO_4_^3−^ and 85% of NH_4_^+^ as nutrients from wastewaters (Govarthanan et al., 2020).

Pharmaceutical wastes are one of the alarming organic pollutants of the environment due to different active compounds like medicines and antibiotics, which cause acute water toxicity (He et al., 2020). To treat organic pollutants present in pharmaceutical wastewater, active groups of polysaccharides in microbial cell walls react with the divalent metal ions present in the waste to form metallic nanoparticles by a cation exchange mechanism. In particular, *Escherichia coli* BL21 isolated from pharmaceutical wastewater produced biogenic palladium nanoparticles by reducing Pd (II) to Pd (0) in the presence of H_2_. Due to high catalytic activity, these nano-sized biogenic palladium particles are reported to reduce and remove more than 87% of ciprofloxacin biotic from wastewaters (He et al., 2020). This might be a unique strategy to treat wastewater by using the micro consortium of waste itself, which hints at greener technology and a circular bioeconomy.

Reactive azo dye, mainly released from the textile industry, is one of the leading causes of water pollution worldwide (Sarkar et al., 2020). Not only EPS alone, as discussed in the previous section, but also EPS mediated biogenic metal nanoparticles degrade dye-containing wastewater, effectively. An electrochemically active biofilm produced by extremophilic bacterial strain *Shewanella loihica* PV-4 isolated from a hydrothermal vent located at Loihi Seamount in the Pacific Ocean has been reported to synthesis ultra-small (2–7nm) palladium and platinum nanoparticles. Remarkably, these nanoparticles showed complete degradation against reactive azo dye like methyl orange within just 50s of incubation (Gao et al., 2006; Ahmed et al., 2018; Gahlawat and Choudhury, 2019).

In another interesting strategy to disinfect and treat industrial wastewater as a bactericidal agent, bio-flocculant EPS produced by marine *Bacillus subtilis* was used for polymeric silver nanoparticles (AgNPs) synthesis. These EPS-stabilized AgNPs were described to have superior flocculating efficacy, high stability (≥5months), and also high bactericidal activity (Sathiyanarayanan et al., 2013). The EPS-AgNP particles significantly reduced the bacterial load in sewage water from the initial ~250 to 7CFU/ml (Sathiyanarayanan et al., 2013). According to Sathiyanarayanan et al. (2017), EPS-based biopolymeric metal nanoparticles have a long shelf life compared to the nanoparticles synthesized using other strategies due to the opposite charge interactions of bacterial EPS and metal ions, which are very accommodating for industrial usage.

As previously mentioned, EPS has been used as a template for the environmentally safe and green synthesis of metallic nanoparticles (Zhang et al., 2020). The NP formation procedure was proposed to be a two-step method, the biosorption of metal ions on the bacterial cell wall or a single EPS, followed by a reduction of these ions resulting in metallic NP formation (Sathiyanarayanan et al., 2016). Besides, EPS can assume a capping agent’s function, maintaining the NP stability by inhibiting their aggregation (Sathiyanarayanan et al., 2017). The presence of single or multiple functional groups of anionic (–COOH, –SO_3_, and –PO_3_), cationic (–NH_2_, –NHCOCH_3_), or neutral (–OH, –CH_2_, –CH_3_, and –CHO) nature could act as a redox pair. In a proposed mechanism, the metal is attracted by functional groups through different attraction forces (e.g., ionic and dipolar) and depending on the pH of the media. This process is followed by an EPS-metal complex formation and further reduction. The metal NP is anchored and stabilized by different functional groups of the EPSs surrounding the metallic particle (Salem and Fouda, 2021). The NPs external charged layer allows particle repulsion giving them physical stability in solution and protection of NPs primary structure in the solid state (Pereira et al., 2015). Indeed, studies exist that demonstrate the ability of EPS to self-assemble and form spherical nanosize particles of ∼88nm in diameter (Li et al., 2017). These self-assembled EPS nanoparticles showed a record biosorption capability for Pb^2+^, Cu^2+^, and Cd^2+^, and methylene blue dye. Such studies expand the manufacturing of novel EPSs and offer a new, eco-friendly, and renewable platform for bioremediation as well as the green synthesis of nanomaterials.

**Figure 2:**
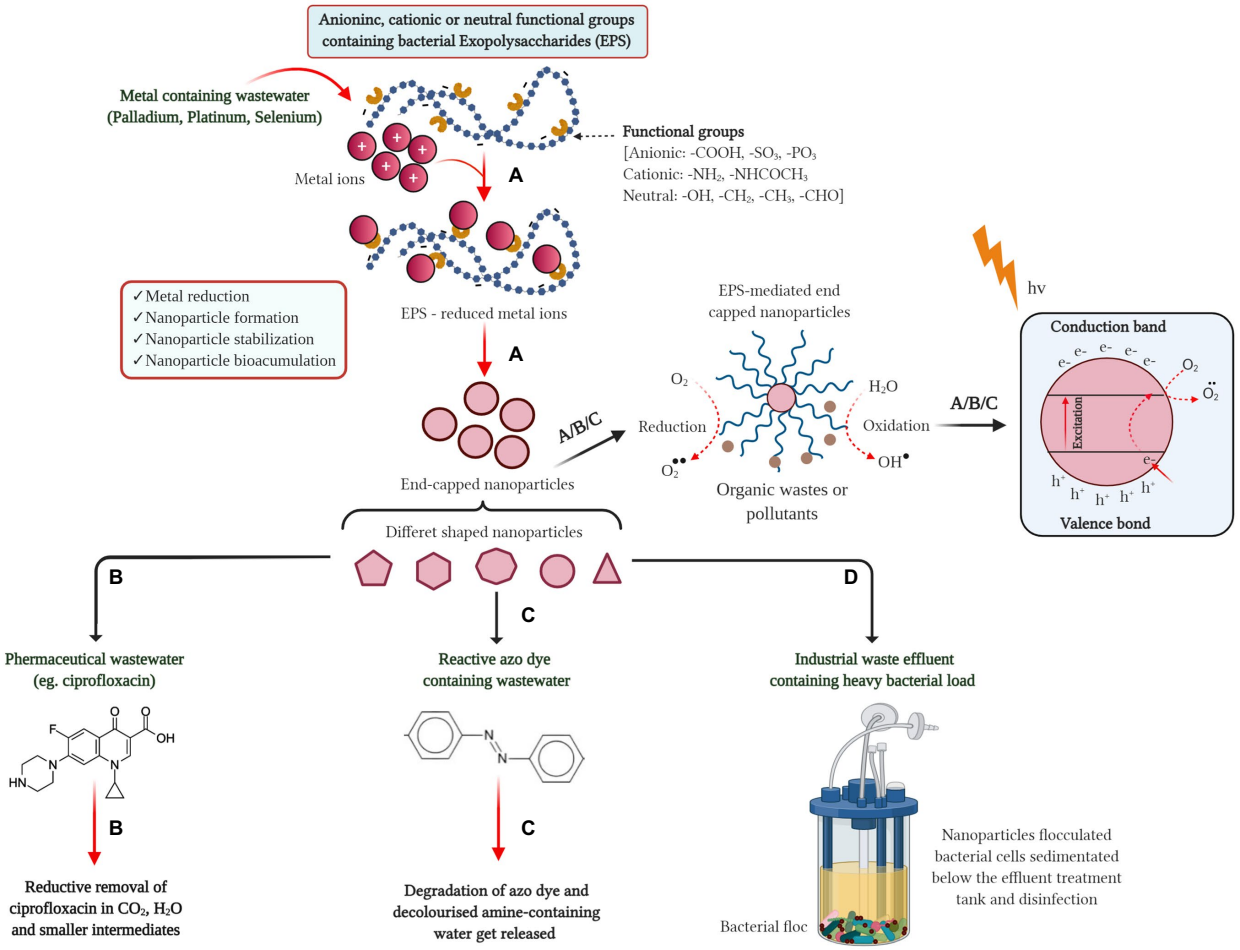
Bacterial EPS in nanoparticle synthesis and its role in separation and adsorption of pollutants from wastewater. **(A)** Metal containing wastewater, **(B)** pharmaceutical wastewater, **(C)** textile wastewater, and **(D)** industrial wastewater with bacterial load.

The overall concept of bacterial EPS-mediated metal nanoparticle synthesis and its role in the separation and adsorption of pollutants from wastewater is summarized in Figure 2. The separation and adsorption of the pollutants from the wastewater occur in a photocatalytic mechanism where contaminated water molecules go through oxidation-reduction in the presence of photons from sunlight (Sarkar et al., 2020), which is also explained in Figure 2. As discussed, biogenic metal nanoparticles exhibited high catalytic activity to transform complex aromatic and organic pollutants or metal wastes into non-toxic molecules in a short reaction time. It is also presumed that biologically developed nanomaterials even have the potential to treat multicomponent water (Gautam et al., 2019). Despite the well-demonstrated applications of extremophilic bacterial EPSs in wastewater bioremediation, the use of bio-flocculants in industrial practice has been inadequate so far. It is not yet commercialized because of the industry-academia gap that needs to be bridged (Kalita and Joshi, 2017). Nevertheless, the special ecological niche of bacteria in extreme environments and their production of structurally diverse EPSs signify that harsh environment throughout the planet harbor a scarcely tapped reservoir of renewable, multifunctional biomaterials for unique industrial applications.

## Conclusion and Future Prospects

Exopolysaccharides are a substantial part of the bacterial biofilm formed in extreme environments to help microbial survival against the deleterious effect of those harsh conditions. They form a connective network surrounding the bacterial cells and enable them to subsist in the otherwise inhospitable environment of extreme temperature, salinity, harsh pH, abundant heavy metals, UV-rays, and more. Due to their non-toxic, biodegradable, and biocompatible nature, biotechnological advantages of the EPSs produced by microbes from extreme environments are more diverse than traditional biopolymers for use in food (for their unusual gelling and thickening properties), textiles (as surfactants in detergents) as well as the cosmeceutical, pharmaceutical, and biomedicine industries (for their immunomodulatory and antiviral effects).

Besides, EPSs possess an extensive array of unusual structural and helpful functional characteristics that have implications for rapid, efficient, sensitive, economical, and high-value applications, such as decontaminate agents in mining and textile industries, among others, and in wastewater treatment involving biosorption, emulsification, and flocculation of textile dyes, ionic wastes, metal ions, heavy metals, oil, hydrocarbons, and more. The bioremediation efficiency of EPS is due to many bioorganic and inorganic compounds like uronic acid, lipids, amino acids, and polysaccharides. In an era of ever-increasing water pollution, climate change, and water scarcity, recycling wastewater becomes a central matter to be solved in the future. EPS is the fundamental solution to defeat the problems associated with the conventional methods of wastewater treatment. Lately, advances have been made using bacterial EPS-stabilized biogenic nanoparticles for application in wastewater treatment. It is anticipated that the practical commercialization of biopolymeric/metal nanomaterials will emerge shortly for industrial effluent treatment and wastewater bioremediation.

However, to be successfully commercialized for usage in industrial wastewater treatment plants, the dosage of metal ions as well as adsorbent (EPS), duration of the interaction, optimum physio-chemical conditions of the bacterial growth, biomass concentration, and red-ox potential of the bacterial EPSs are some of the factors that need to be stringently regulated and controlled, to yield maximum effectiveness of the bioremediation process (Gupta and Diwan, 2017). The primary deciding factor that lies here is the reusability and regeneration of the EPS, the maximum limits of which are generally limited. EPS to be used for bioremediation must be used repeatedly (Wang et al., 2013). Also, it has been evident that the sorption through EPS is generally highly dependent on its anionic character. If the EPS has less percentage of anionic groups attached to it, and then that decreases the biosorption capacity of the EPS. Moreover, with many environmental controlling factors, such as temperature, pH, and ionic strength, capable of affecting the strength of EPS, strict regulation, and control of decisive factors on an industrial scale is often difficult.

To be successfully scaled up from lab to commercial scale wastewater treatment, EPS immobilized or attached on suitable carriers can be a savior here. Also, mining high anionic polymer yielding strains should be helpful. Since EPS is a non-living entity, its usage for detoxification/bioremediation of the environment is free from the pathogenicity issues associated with the use of whole organisms (Díaz et al., 2018). In the future, it would be helpful to combine the role of EPS usage with other modern wastewater treatment technologies; these include ultra/nanofiltration, reverse osmosis, electrodialysis, magnetic nanomaterials, and microbial fuel cells. It is a crucial issue to find a niche application, given that industrial effluents are more complex than laboratory samples and that several pollutants, in addition to the primary target product, are present (Li et al., 2017). In any case, more profound knowledge of the structure-function relationships of the bacterial EPSs can be expected to open up further possibilities for harnessing the full potential of these exceptional biomaterials. Finally, industrial effluent studies, production cost determination, and pilot-scale trials will succeed in the commercial utilization of extremophile EPSs in wastewater remediation.

## Author Contributions

AB and RKS: conceptualization. AB, SS, and TG: writing – original draft preparation. SS, PG-F, AB, and TG: figure preparation. AB, GC-B, SS, RB, DRS, and RKS: writing – review and editing. RKS: supervision. All authors contributed to the article and approved the submitted version.

## Conflict of Interest

The authors declare that the research was conducted in the absence of any commercial or financial relationships that could be construed as a potential conflict of interest.

## Publisher’s Note

All claims expressed in this article are solely those of the authors and do not necessarily represent those of their affiliated organizations, or those of the publisher, the editors and the reviewers. Any product that may be evaluated in this article, or claim that may be made by its manufacturer, is not guaranteed or endorsed by the publisher.

## Footnotes

^1^ https://relecura.com/

## Supplementary Material

Supplementary Figure 1 Various applications of microbial exopolysaccharides (EPS) in biotechnology.

Supplementary Figure 2 Overview on EPS producing extremophiles.
