# Supplementary figures and images for: Extremophilic Exopolysaccharides: Biotechnologies and Wastewater Remediation
Source: Front Microbiol. 2021 Aug 19;12:721365. doi: 10.3389/fmicb.2021.721365 (PMC8417407; doi:10.3389/fmicb.2021.721365)

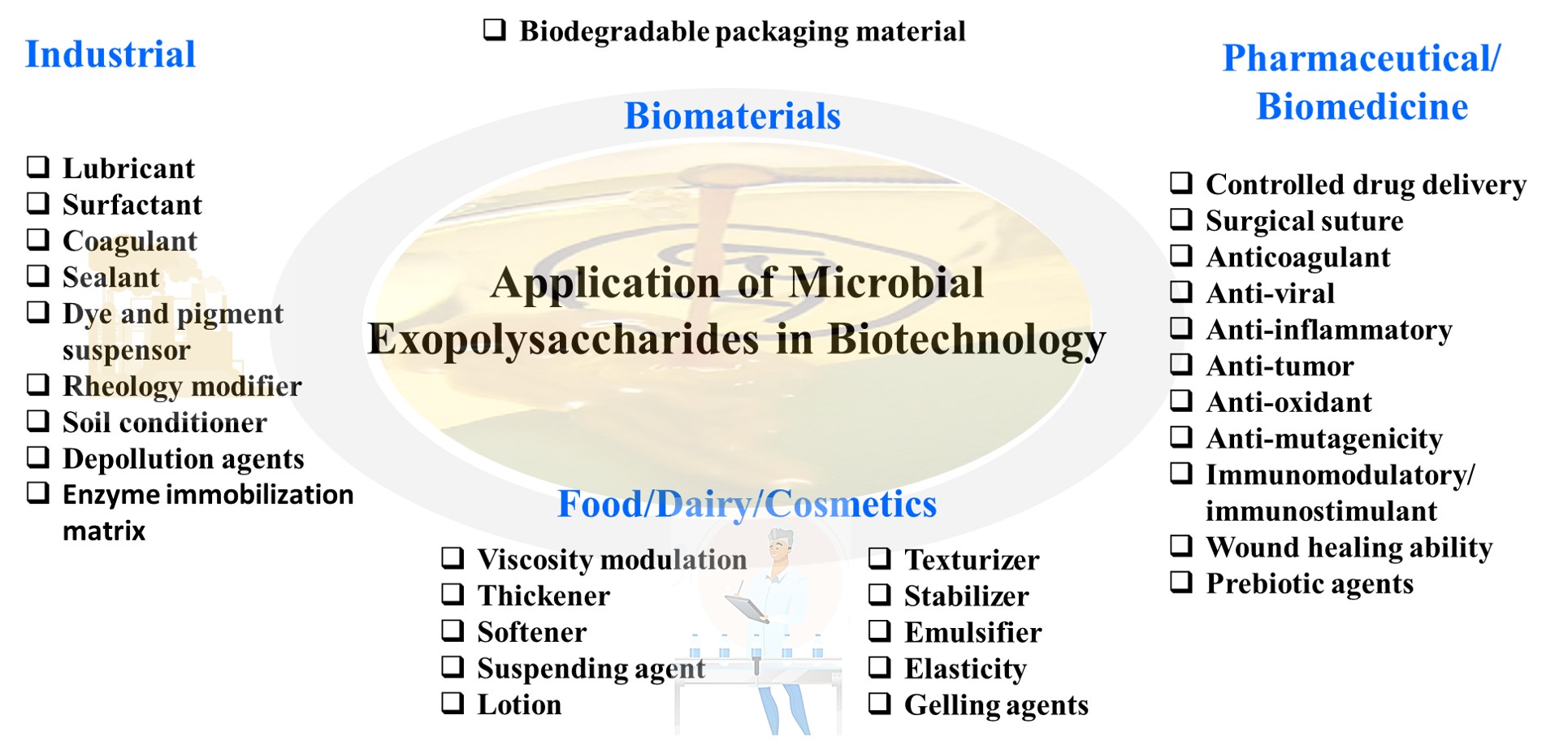

Supplement: Supplementary Figure 1 — Various applications of microbial exopolysaccharides (EPS) in biotechnology. [file Image_1.jpeg]

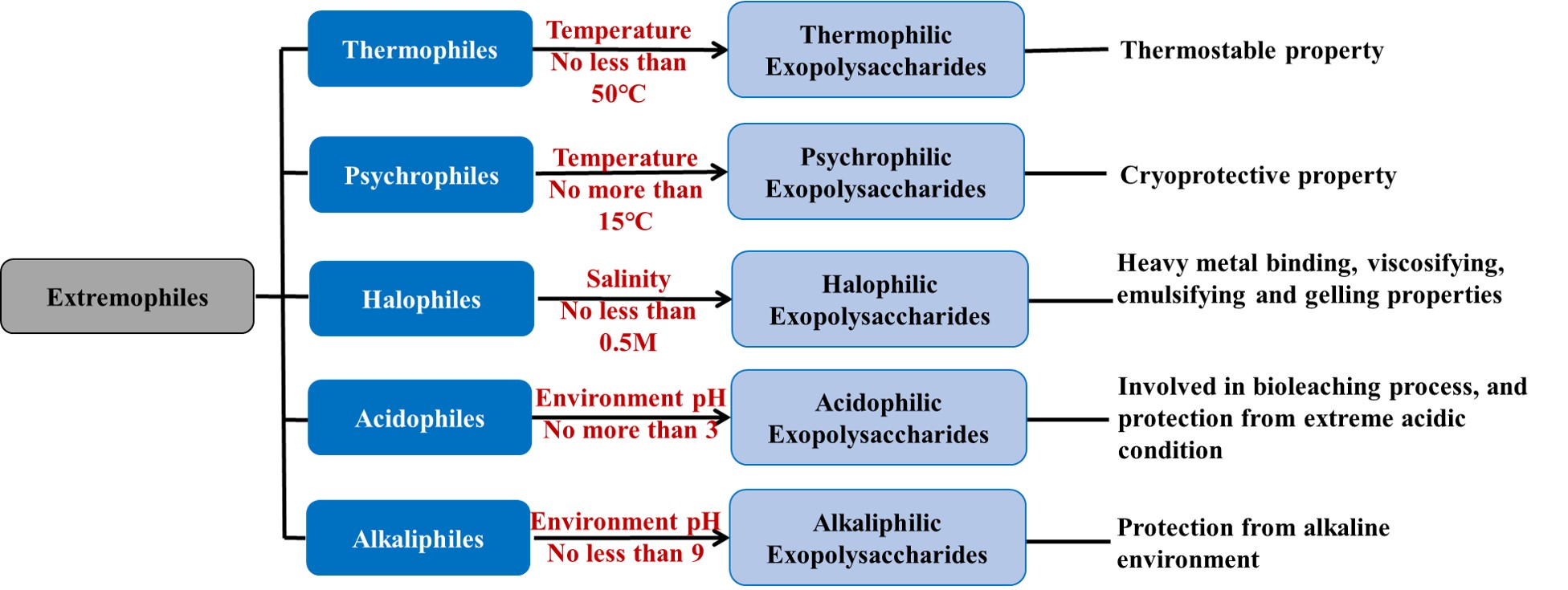

Supplement: Supplementary Figure 2 — Overview on EPS producing extremophiles. [file Image_2.jpeg]
